# Safety of Special Waveform of Transcranial Electrical Stimulation (TES): In Vivo Assessment

**DOI:** 10.3390/ijms23126850

**Published:** 2022-06-20

**Authors:** Muhammad Adeel, Chun-Ching Chen, Bor-Shing Lin, Hung-Chou Chen, Jian-Chiun Liou, Yu-Ting Li, Chih-Wei Peng

**Affiliations:** 1 School of Biomedical Engineering, College of Biomedical Engineering, Taipei Medical University, Taipei 110, Taiwan; d845108004@tmu.edu.tw (M.A.); jcliou@tmu.edu.tw (J.-C.L.); 2 International PhD Program in Biomedical Engineering, College of Biomedical Engineering, Taipei Medical University, Taipei 110, Taiwan; 3 Department of Interaction Design, College of Design, National Taipei University of Technology, Taipei 106, Taiwan; cceugene@ntut.edu.tw; 4 Department of Computer Science and Information Engineering, National Taipei University, New Taipei City 237, Taiwan; bslin@mail.ntpu.edu.tw; 5 Department of Physical Medicine and Rehabilitation, School of Medicine, College of Medicine, Taipei Medical University, Taipei 110, Taiwan; 10462@s.tmu.edu.tw; 6 Department of Physical Medicine and Rehabilitation, Shuang Ho Hospital, Taipei Medical University, New Taipei City 235, Taiwan; 7 Taiwan Instrument Research Institute, National Applied Research Laboratories, Hsinchu 30261, Taiwan; ytl@tiri.narl.org.tw; 8 School of Gerontology Health Management, College of Nursing, Taipei Medical University, Taipei 110, Taiwan

**Keywords:** intermittent theta burst stimulation (iTBS), transcranial direct current stimulation (tDCS), electrical stimulation, safety parameters, primary cortex, in vivo, current density, duration, frequency, scalp

## Abstract

Intermittent theta burst (iTBS) powered by direct current stimulation (DCS) can safely be applied transcranially to induce neuroplasticity in the human and animal brain cortex. tDCS-iTBS is a special waveform that is used by very few studies, and its safety needs to be confirmed. Therefore, we aimed to evaluate the safety of tDCS-iTBS in an animal model after brain stimulations for 1 h and 4 weeks. Thirty-one Sprague Dawley rats were divided into two groups: (1) short-term stimulation for 1 h/session (sham, low, and high) and (2) long-term for 30 min, 3 sessions/week for 4 weeks (sham and high). The anodal stimulation applied over the primary motor cortex ranged from 2.5 to 4.5 mA/cm^2^. The brain biomarkers and scalp tissues were assessed using ELISA and histological analysis (H&E staining) after stimulations. The caspase-3 activity, cortical myelin basic protein (MBP) expression, and cortical interleukin (IL-6) levels increased slightly in both groups compared to sham. The serum MBP, cortical neuron-specific enolase (NSE), and serum IL-6 slightly changed from sham after stimulations. There was no obvious edema or cell necrosis seen in cortical histology after the intervention. The short- and long-term stimulations did not induce significant adverse effects on brain and scalp tissues upon assessing biomarkers and conducting histological analysis.

## 1. Introduction

Among the non-invasive neuromodulation approaches, transcranial magnetic (TMS) and direct current stimulations (DCS) are used to regulate cortical excitability [1,2]. Clinically, both stimulation techniques can induce neuroplasticity in improving motor and memory functions in patients [3,4,5,6]. Transcranial magnetic stimulation (TMS) can be applied in the form of continuous, repetitive, or burst waveforms through a magnetic coil over the head that works by generating an electric field in the brain via electromagnetism [7]. A special form of rTMS consists of a series of short bursts at a high inner frequency interspersed with brief periods of no stimulation, called theta-burst stimulation (TBS). It consists of short bursts of 50 Hz rTMS which are repeated at a rate in the theta range (5 Hz) as a continuous (cTBS) or intermittent (iTBS) train, and is the most widely utilized method [8,9]. One commonly used transcranial electrical stimulation (TES) is a transcranial DCS (tDCS), which utilizes large (25–35 cm^2^) electrodes to provide a small amount of direct current (1–2 mA) to the scalp [10,11]. The stimulation alters brain function by depolarizing or hyperpolarizing the resting membrane potential of the cell. The current used in anodal tDCS depolarizes the resting membrane potential, increasing neuronal firing and producing long-term potentiation (LTP)-like effects. Cathodal tDCS induces a long-term depression (LTD)-like effect, which causes the resting membrane potential to become hyperpolarized. Because of reduced impulsive cell firing, this LTD-like effect reduces neuronal excitability [2,12]. 

According to Lisman and Idiart, theta-frequency oscillations regulate high-frequency gamma oscillations, which are related to cognitive processing in human recognition memory [13]. Furthermore, iTBS raises the amplitude of subsequent I-waves and generates LTP-like alterations at synaptic junctions in the motor cortex by altering the intrinsic circuitry of the motor cortex [8,14]. Furthermore, tDCS generates plastic after-effects via membrane polarization and NMDA receptor-mediated glutamatergic synaptic transmission, according to several human and animal investigations [15,16]. Previous studies reported that the iTBS-like anodal DC stimulation elicited the two mentioned neuroplastic processes of iTBS and tDCS at the same time, resulting in a cumulative impact on neuroplasticity. In comparison to traditional tDCS, it was found that iTBS-like anodal DC stimulation caused a larger amplitude of motor evoked potential (MEP) [7]. 

A previous study reported the use of 10 Hz and iTBS dorsomedial prefrontal cortex (DMPFC) rTMS as a safe and tolerable stimulation intervention for major depression. The outcomes were compared for 6 min iTBS and 30 min 10 Hz protocols in a clinical population [17]. In recent years, TES—specifically tDCS—has received more attention as a potential therapy for neurological and psychiatric illnesses. Several attractive clinical properties, including safety, tolerability, the convenience of use, affordability, and portability, have piqued interest in employing TES for therapeutic purposes [10]. It has been hypothesized that daily anodal tDCS across the left dorsolateral prefrontal cortex (DLPFC) relieves depression by reversing the hypoactivity found in major depressive disorder (MDD) [18]. One study proposed that depression is linked to lower levels of brain-derived neurotrophic factor (BDNF), a neurotrophin important for synaptic strengthening and neuronal endurance [19], and suggested that the upregulation of BDNF levels, as a critical neurobiological mechanism for depression alleviation, might be involved in antidepressant actions. 

Based on simulated and animal studies, only a percentage of the electric current penetrates the cortex, resulting in neuronal polarization and excitability in the cortex [20,21,22,23] and hippocampus [20,24]. tDCS has been studied for many clinical and assistive applications, including depression [25,26], motor rehabilitation [27], speech rehabilitation [28,29,30], pain control [31,32,33], and working memory [34]. Regarding safety, tDCS was used in previous studies without any adverse effects when appropriate standards were implemented [10,35,36,37,38,39]. Currently, the use of tDCS across many applications is widespread, so more studies are required to standardize the safe tDCS dosing parameters [40,41,42]. Few studies have evaluated the effects of the special waveform of tDCS, i.e., iTBS. Conventionally, iTBS is outputted from TMS to induce neuroplasticity in nerve tissue [43,44]. However, recently, two studies by our search team reported the use of iTBS waveform delivered through electrical stimulations in animals in vivo experiments [7] and stroke patients [45] for its efficacy. In an animal study, it was reported that tDCS-iTBS (0.25 mA tDCS and 50 Hz iTBS for 190 s) is a feasible therapeutic approach for enhancing neuromodulatory effects [7], while the clinical study implied that the stroke patients had significantly improved upper limb functioning from using tDCS-iTBS (1 mA anodal tDCS and 1.5 mA iTBS for 20 min for 3 days/week for 6 weeks) stimulation along with rehabilitation compared to rehabilitation alone [45].

To assess the safety of tDCS on microglial cells in rodents, one study reported the activation of these cells by 0.5 mA anodal or cathodal stimulation for 15 min [46]. Another study showed that anodal tDCS at a current intensity of 0.4 mA (31.8 A/m^2^ electrode current density) induced microglial alterations in neurodegeneration-related morphology [47]. The histological analysis of one study mentioned that anodal tDCS can induce brain lesions in rats at a stimulation intensity of 0.5 mA using 25 mm^2^ electrodes (20 A/m^2^) [48]. Liebetanz et al. reported the brain lesion threshold intensity for cathodal tDCS as 0.001–1 mA for 15–270 min (143 A/m^2^) [49]. The safety effects of tDCS on the human brain were studied by using neuron-specific enolase (NSE), magnetic resonance imaging (MRI), and electroencephalography (EEG) data in previous studies [15,50,51]. There is a scarcity of research on the safety of special waveform of tDCS, i.e., iTBS, in animal cortex using biomarkers and immunohistological assays. One of the studies examined the effects of deep brain stimulation (DBS) on apoptosis in the hippocampal pedunculopontine tegmental nucleus (PPTg). It reported a decreased level of caspase-3 for apoptotic activity and myelin basic protein (MBP) for brain injury biomarkers, without any change in cytokine interleukin-6 (IL-6) for inflammation after the application of DBS [52]. According to the analysis of cortical activity markers, the expressions of GABA-synthesizing enzyme GAD67 (67 kD isoform of glutamate decarboxylase), calcium-binding proteins parvalbumin (PV), and calbindin (CB) were lowered by iTBS but not by cTBS one day after the last session. Because the magnetic stimulation did not target a specific cortical location, these alterations were visible in many cortical areas in all animals, regardless of whether they completed the task. However, the frontal and barrel cortex, which are engaged in the learning process, exhibited a considerably smaller reduction in PV and CB expression than the visual cortex, which was not involved in the activity [53].

Until the present research, no study has reported the neuroprotective effects of the special waveform of tDCS, i.e., iTBS, on a rat brain after short- and long-term stimulations using a low current density. Therefore, the rationale of this study was to evaluate the safety of the iTBS waveform delivered through electrical current power by the previously designed and implemented novel transcranial burst electrostimulation device, which confirmed the feasibility and neuroplastic effects of tDCS-iTBS waveform for neurostimulation on the brain [7] in vivo experiments. Thus, in our current study, we conducted brain and scalp tissue analysis to evaluate the safety of the tDCS-iTBS in the animal model after stimulations for 1 h and 4 weeks.

## 2. Results

The results of the present study demonstrate a non-significant change in biomarkers (caspase-3 activity, cortical and serum MBP levels, NSE expression, and cortical and serum IL-6 levels) and histology of cortical tissues after short- and long-term stimulations to highlight the safety of tDCS-iTBS waveform intervention (Table 1).

### 2.1. Effect of tDCS-iTBS on Caspase-3 Activity

**Figure 1:**
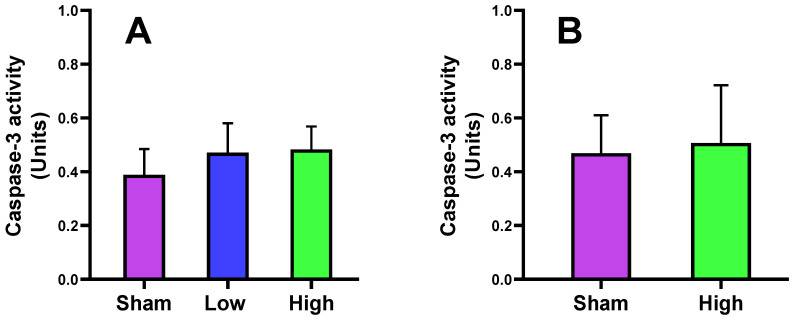
Effect of tDCS-iTBS on caspase-3 activity: (**A**) short-term (*n* = 15) and (**B**) long-term stimulations (*n* = 16).

In this study, we found that caspase-3 activity increased in low (2.5 mA/cm^2^) and high current density (4.5 mA/cm^2^) interventions in short- and long-term stimulations compared to sham. The high-density stimulation resulted in slightly more increased activity of caspase-3 in the rat cortex. The difference between sham, low, and high current density for short- and long-term stimulations did not reach a significance level (*p* values > 0.05, Figure 1).

### 2.2. Effect of tDCS-iTBS on Brain and Skin/Serum Biomarkers

#### 2.2.1. Cortical MBP Expression

**Figure 2:**
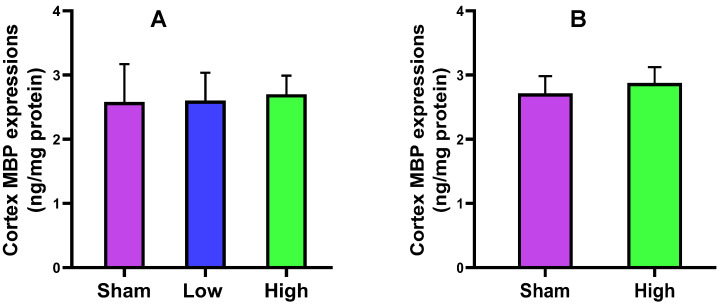
Effect of tDCS-iTBS on cortical MBP expressions (ng/mg protein): (**A**) short-term (*n* = 15) and (**B**) long-term stimulations (*n* = 16).

The myelin basic protein (MBP) is the most abundant protein of myelin in oligodendrocytes in the central nervous system (CNS) and Schwan cells in the peripheral nervous system (PNS). It is responsible for the myelination of neurons. In this study, the cortical expression of MBP (ng/mg) was increased in both short- and long-term stimulation groups on high current density interventions. None of the groups attained the level of significance (*p* values > 0.05, Figure 2). 

#### 2.2.2. Serum MBP Expression

**Figure 3:**
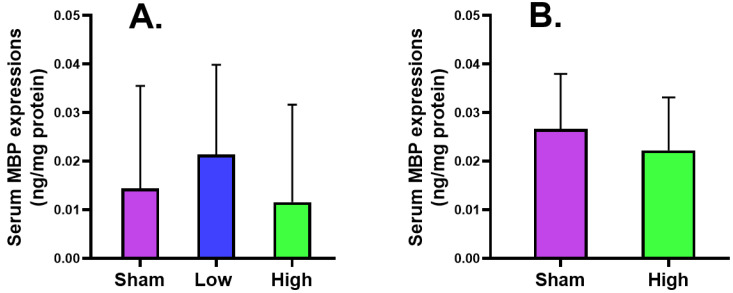
Effect of tDCS-iTBS on serum MBP expressions (ng/mg protein): (**A**) short-term (*n* = 15) and (**B**) long-term stimulations (*n* = 16).

The serum MBP expression after short-term stimulation was higher in low current density intervention but lower in high current density intervention than sham for both short- and long-term stimulation groups without reaching a significant level (Figure 3).

#### 2.2.3. Cortical NSE Expression

**Figure 4:**
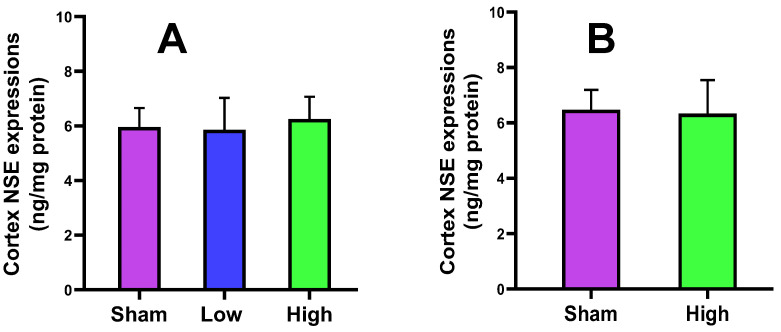
Effect of tDCS-iTBS on cortex NSE expressions (ng/mg protein): (**A**) short-term (*n* = 15) and (**B**) long-term stimulations (*n* = 16).

The neuron-specific enolase (NSE) is a prominent biomarker of ischemic brain injury. By the application of short-term stimulation, NSE expression was higher in high current intervention but did not change in low current intervention as compared to the sham. For long-term stimulation, no change in NSE expression was observed from the sham. For both groups, no intervention attained the level of significance (Figure 4). 

#### 2.2.4. Cortical IL-6 Levels

**Figure 5:**
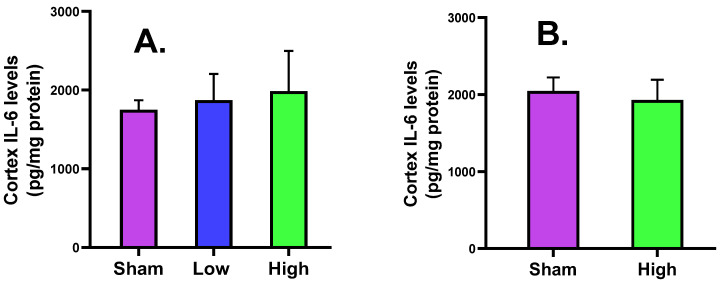
Effect of tDCS-iTBS on cortical IL-6 levels (pg/mg protein): (**A**) short-term (*n* = 15) and (**B**) long-term stimulations (*n* = 16).

The cortical IL-6 was increased for high current density rather than low current density intervention during short-term stimulation, and was decreased for high current density compared to sham for long-term stimulation, without any significant difference (Figure 5).

#### 2.2.5. Serum Tissue IL-6 Levels

**Figure 6:**
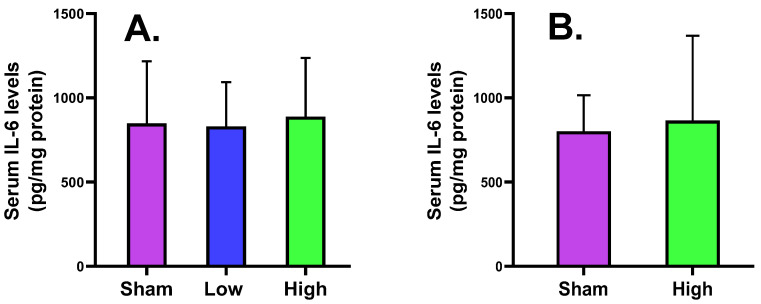
Effect of tDCS-iTBS on serum IL-6 levels (pg/mg protein): (**A**) short-term (*n* = 15) and (**B**) long-term stimulations (*n* = 16).

The scalp skin tissue pro-inflammatory biomarker IL-6 level showed that high current intervention increased the IL-6 levels for both short- and long-term stimulation groups, but low current intervention decreased for short-term stimulation. There is no statistically significant difference between the three interventions, suggesting that high current density can affect the scalp tissue under-stimulation more than low current intervention (Figure 6).

### 2.3. Effect of tDCS-iTBS on Brain Tissue Morphology (H&E Staining)

#### 2.3.1. Short-Term Stimulation H&E Staining

**Figure 7:**
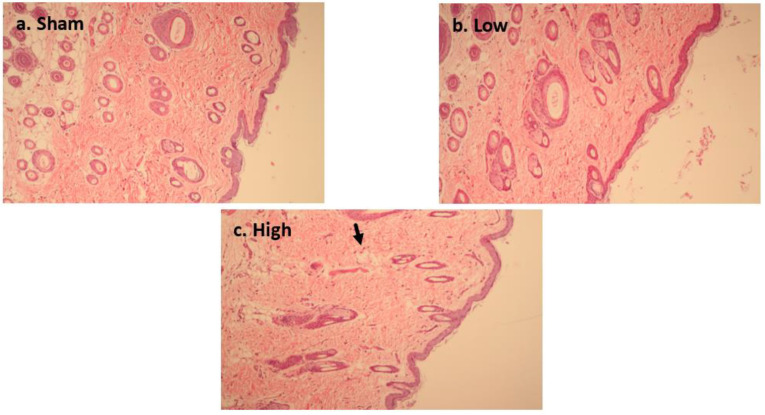
Effect of tDCS-iTBS on cortical tissue morphology (H&E staining) during short-term stimulation (*n* = 15); (**a**) sham stimulation, (**b**) low-intensity stimulation (2.5 mA/cm^2^), and (**c**) high-intensity stimulation (4.5 mA/cm^2^).

Figure 7a–c presents cortical tissue histology using H&E staining. After applying short-term stimulation, only high current density intervention (Figure 7c) showed mild edema without obvious cell shrinkage, necrosis, or microglial changes. Low current density intervention (Figure 7b) did not show any change from the sham intervention (Figure 7a). These results reflect the safety of short-term stimulations on rat cortical tissue because there was no obvious difference in tissue morphology after either intervention. 

#### 2.3.2. Long-Term Stimulation Staining

**Figure 8:**
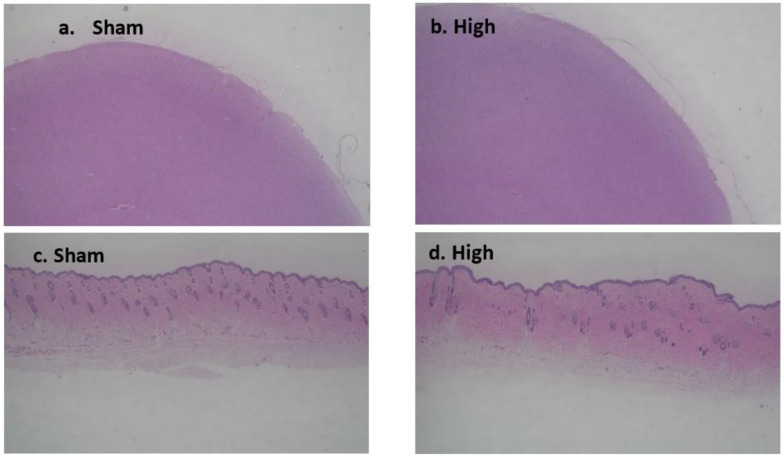
Effect of tDCS-iTBS on cortical tissue morphology (H&E staining) during long-term stimulation (*n* = 16); (**a**) sham and (**b**) high-intensity stimulation (4.5 mA/cm^2^) subgroups 40 µm coronal tissue section, while (**c**) sham and (**d**) high-intensity stimulation (4.5 mA/cm^2^) magnified tissue section to represent stimulation efffect.

During long-term stimulation, no obvious change was observed in H&E staining, except for a mild decrease in the number of neuron cells in high current density intervention. There was no clear difference from sham intervention, and no edema, shrinkage, or necrosis was observed (Figure 8a–d). 

## 3. Discussion

In the present study, we report the effects of the special waveform of tDCS, i.e., iTBS, which is electrically powered as opposed to conventional magnetic stimulation. For the two groups including short- and long-term stimulations, we did not find any significant effect of the low and high current density on the rat cortex and scalp skin tissues. Based on the biomarker analysis and histological staining, it is thus confirmed that tDCS-iTBS in the range of 2.5 to 4.5 mA/cm^2^ did not induce any brain and skin damage because the current density is below the lesion threshold of 14.2 mA/cm^2^ [49], ensuring the safety of tDCS-iTBS waveform for use in animals to induce neuroplasticity. 

The safety of the tDCS-iTBS waveform was evaluated after short- and long-term stimulations. The caspase-3 activity of the rat cortex was not significantly changed in either group. For short-term stimulation, caspase-3 activity did not obviously increase (sham = 0.39 ± 0.10, low = 0.47 ± 0.11, and high = 0.48 ± 0.08), and the same effect was noted for long-term stimulation (sham = 0.47 ± 0.14 and high = 0.51 ± 0.21). One previous study reported the effect of tDCS on caspase-3 activity after middle cerebral artery occlusion in ischemic stroke rats. They observed that cathodal tDCS can significantly reduce caspase-3 expression (*p* = 0.000 *), which is raised after ischemic injury, and inhibit apoptosis at the injury site [54]. Based on our study results, after applying the different intensity tDCS-iTBS stimulations, the protein caspase-3 activity did not significantly differ from the sham. 

The cortical MBP level was slightly increased for low (2.62 ± 0.44) and high (2.71 ± 0.27) current density compared to sham (2.57 ± 0.57) in the short-term stimulation group, and also slightly increased for high (2.87 ± 0.25) current density compared to sham (2.71 ± 0.27) in the long-term stimulation, without a significant difference. The serum MBP level slightly increased for only low (0.02 ± 0.02) current density compared to sham (0.01 ± 0.02) in the short-term stimulation, but a decreasing trend was noted for high (0.02 ± 0.01) current density compared to sham (0.03 ± 0.01) in the long-term stimulation. The NSE expression slightly decreased for low current density (5.86 ± 1.16) and increased for high current density (6.26 ± 0.80) compared to sham (5.96 ± 0.69) in the short-term stimulation, while it decreased for high current density (6.33 ± 1.21) compared to sham (6.48 ± 0.72) in the long-term stimulation. The cortical IL-6 level was increased for low (1871.44 ± 332.47) and high (1985.92 ± 511.68) current density compared to sham (1748.81 ± 122.30) in the short-term stimulation group but slightly decreased for high (1930.00 ± 263.71) current density compared to sham (2046.38 ± 175.93) in the long-term stimulation, without a significant change. The serum IL-6 level slightly decreased for low current density (830.49 ± 263.27) and increased for high current density (888.29 ± 347.84) compared to sham (848.71 ± 368.95) in the short-term stimulation, but an increase was noted for high (865.75 ± 502.56) current density compared to sham (801.63 ± 213.89) in the long-term stimulation. The histology of brain tissues of both groups reported no significant difference from sham in terms of edema, cell shrinkage, and necrosis, confirming the safety of the tDCS-iTBS waveform in the present study. 

Based on recent research, tDCS protocols can be tolerable and safe if well-defined electrodes, stimulus durations, and intensities are employed [10]. The tDCS application is determined by its tolerability and safety. Tolerability means the presence of unpleasant and unexpected effects (such as tingling and itching sensation below the electrodes), while safety implies harmful effects. Overall, the presently used tDCS profiles are tolerable [55,56]. tDCS may cause erythema under the electrodes due to vasodilation, which is not a safety concern [57]. The safety is determined strictly by structural brain tissue damage, as reported by previous research [15,58]. Skin damage is occasionally reported in other studies [59] but mostly occurs due to the drying of contact media under the electrodes. The use of tap water which can induce skin burns or electrode gel is not appropriate, so electrode cream is recommended between the skin and electrode surface [60,61].

The stimulation current density used in this study ranged from 2.5 to 4.5 mA/cm^2^ (tDCS: 1–3 mA; iTBS: 1.5 mA) applied for 1 h during short-term stimulations and 30 min during long-term stimulations. Previously, a study by Bikson et al. (2016) reported the safety of tDCS for 33,000 sessions on 1000 patients who received tDCS. Their report mentioned that there was no serious adverse effect on the brain by using conventional tDCS in clinical trials (≤40 min, ≤4 mA, and ≤7.2 C) [41]. The parameters for stimulation in our study spanned were 30 min–1 h, 1–3 mA tDCS combined with 1.5 mA iTBS for each subgroup, charge of 8.1–16.2 C, and current density of 2.5–4.5 mA/cm^2^. Three research groups mentioned the brain injury threshold for tDCS application: (1) Liebetanz et al., 14.2 mA/cm^2^ (used 500 µA current with 3.5 × 3.5 mm electrodes for 10 min) [49]; (2) Fritsch, applied 600 µA through 4 mm diameter electrode for 20 min; and (3) Jankord, through 500 µA using 5 × 5 mm electrodes for 60 min [41]. 

To assess the safety of the tDCS-iTBS waveform, there are only a few studies available. One study utilized anodal tDCS-iTBS waveform on rat cortex and found significant neuroplastic effects (35% and 100% increase in MEP amplitude to induce LTP-like effects) than traditional tDCS waveform. They used iTBS in the range of 0 to 1.5 mA (0.25 mA/step) combined with tDCS of 0.25 mA for 190 s and did not report any adverse effects [7]. Another study applied 1 mA tDCS combined with 1.5 mA iTBS for 20 min on the primary motor cortex of stroke patients and found a significant improvement in upper limb recovery without adverse events [45]. Based on the available research, one study reported a scaling factor to determine the threshold damage of tDCS current application from rats to humans as 173 mA from Fritsch, 120 mA from Liebetanz, and 67 mA from Jankord [41]. 

Regarding the limitations of this study, (1) the safety of the tDCS-iTBS waveform was tested on only animals. Future studies should be conducted to test the safety of the tDCS-iTBS waveform on humans to start the clinical application to treat neurological disorders such as depression, stroke, spinal cord injury, or Alzheimer’s disease. (2) A limited number of animals were tested in the present study; a large sample size in different subgroups should be considered for the reproducibility of results in future research. (3) Safety on the brain and scalp should be determined through EEG or MRI-based studies using the same waveform in future research.

## 4. Materials and Methods

### 4.1. Animal Handling and Tissue Extraction 

#### 4.1.1. Selection and Care of Animals

The study included 31 (*n* = 31) adult male Sprague Dawley rats with weights ranging from 200 to 260 g from BioLASCO Taiwan, Yilan, Taiwan. According to ethical guidelines, all of the rats were kept in a well-equipped animal facility in a sterile, temperature- and humidity-controlled setting. All of the rats were housed on a 12:12 h light:dark cycle with unlimited access to pellet nutrition and water ad libitum. The animals were accustomed for 7 to 10 days before being used in the study, and the experimental procedures and animal use/methods were authorized by Taipei Medical University’s Institutional Animal Care and Use Committee (IACUC-TMU approval no. LAC 2016-0316, 2017-01-01).

#### 4.1.2. Grouping and Experiment Schedule of Animals

All the study animals were categorized into two groups: (1) short-term stimulation (*n* = 15) and (2) long-term stimulation (*n* = 16). The animals in the short-term stimulation group were divided into three subgroups, each containing 5 animals: sham, low, and high current intensity tDCS-iTBS. The animals in the long-term stimulation group were divided into two subgroups, with each having 8 animals: sham and high current intensity tDCS-iTBS. By applying tDCS-iTBS stimulation, the rat brain and scalp skin tissues were extracted after 24 h for group 1 and after 4 weeks for group 2. 

#### 4.1.3. tDCS-iTBS Brain Stimulation Protocol

The tDCS-iTBS intervention was applied through an electrostimulation device which produces varied current intensity ranging from 0 to 3 mA tDCS and from 0 to 1.5 mA iTBS (0.25 mA/step) [7]. Before stimulation, each rat was given urethane anesthesia (1.2 g/kg). The two disposable electrodes (Medihightec Medical Company, Keelung, Taiwan) were placed at the same location for both groups [62]: an active anode (1 cm × 1 cm) over the right skull area on the primary motor cortex of the right forelimb concerning functional brain mapping already carried out in rat model [63], and a reference electrode (5 cm × 3 cm) over an abdomen. 

The applied iTBS waveform output from tDCS (electric power) consisted of a 2 s train repeated after 10 s for 20 cycles (190 s, total 600 pulses). The frequency of each burst was 50 Hz, with three pulses of each 5 Hz repeated every 200 ms [9,64]. For the short-term stimulation, subgroups received (1) sham 0 mA tDCS-iTBS, (2) low 1 mA tDCS + 1.5 mA iTBS (2.5 mA/cm^2^), and (3) high 3 mA tDCS + 1.5 mA iTBS (4.5 mA/cm^2^) for 1 h for a single session. Long-term stimulation subgroups received (1) sham 0 mA tDCS-iTBS and (2) high 3 mA tDCS + 1.5 mA iTBS (4.5 mA/cm^2^) for 30 min per day, 3 days a week for a total of 4 weeks (Table 2).

#### 4.1.4. Sacrifice, Removal of Brain Samples, and Brain and Skin Tissue Collection

Animals were sacrificed using an overdose of urethane (4 g/kg) by cardiac puncture to extract the brain tissue. After being sacrificed, animals were beheaded using a scissor, and their brains were gently extracted. Then, the brain and skin tissues were rinsed in cold isotonic saline before being blotted on filter paper [65]. The brain tissue from the cortex and skin tissue were collected and rinsed again with saline. Excess saline was dried with absorbent paper, and the brain and skin tissues were frozen at −80 °C. The tissues were then homogenized in saline (10% *w*/*v*) at 4 °C. The homogenous specimens were spun at 3000 rpm for 10 min in a refrigerating centrifuge, and the supernatant was collected for examination [52]. Cortical apoptotic activity through caspase-3, cortical and serum MBP expression, cortical NSE, and cortical and serum IL-6 levels were analyzed.

### 4.2. Biochemical Analysis

#### 4.2.1. Evaluation of Caspase-3 Activity

A caspase-3 colorimetric assay kit (Biovisiom, Milpitas, CA, USA) was utilized to measure caspase-3 activity. First, 50 µL of brain tissue specimen was added to a 96-well plate, followed by 50 µL of 2× reaction buffer (containing 10 mM DTT) and 5 µL of the 4 mM DEVD-pNA substrate. The plates were then put into an incubator at 37 °C for 1–2 h, and the absorbance was determined in an ELISA reader at 405 nm [52].

#### 4.2.2. Enzyme-Linked Immunosorbent Assay (ELISA)

The levels of MBP, NSE, and IL-6 were calculated through ELISA kits (Duo-Set; R&D Systems Inc., Minneapolis, MN, USA). The tissue specimens were placed into an incubator for 2 h with a biotinylated rabbit antibody, followed by the addition of streptavidin-conjugated horseradish peroxidase for 20 min. 3,3′,5,5′-tetramethylbenzidine/H_2_O_2_ (R&D Systems Inc., Minneapolis, MN, USA) was added for 30 min, which started the peroxidase reaction, and 0.5 M H_2_SO_4_ stopped it. The absorbance was recorded at 450 nm [52].

### 4.3. Histological Analysis

**Figure 9:**
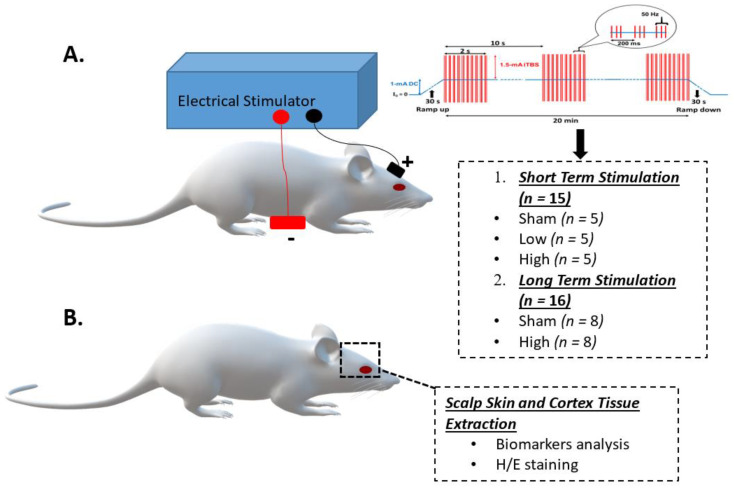
Schematic representation of (**A**) electrical stimulation of rat primary cortex; (**B**) scalp skin and cortex tissue extraction after short- and long-term stimulation interventions.

The 40 µm thick coronal section of brain tissues was processed for hematoxylin and eosin (H&E) staining. The histology of tissues was assessed through light microscopy for pathological changes such as edema, necrosis, and hematoma, as described in the literature on electrical neurotrauma [49,66] (Figure 9).

### 4.4. Statistical Analysis

The study data are reported using mean ± standard deviation (SD) and one-way analysis of variance (ANOVA) for comparing inter-group intervention for cortical caspase-3 activity, cortical and serum MBP expression, cortical NSE expression, and cortical and serum IL-6 levels. The level of significance was set to a *p*-value < 0.05. GraphPad Prism 6 software (GraphPad Software, San Diego, CA, USA) was used for statistical analysis.

## 5. Conclusions

The safety of tDCS-iTBS can be ensured using electrode placement (animal: active electrode on brain and reference electrode on abdomen; human: active electrode on head and reference on scalp), electrode size (animal: 1 cm × 1 cm; human: 5 cm × 5 cm), electric current penetration into the brain (animal: 1.5 mm thick skull; human: 6–7 mm thick skull), and current density (animal: cathodal tDCS = 142.9 A/m^2^, anodal tDCS = 20 A/m^2^ (our study = 25–45 A/m^2^); human tDCS: 0.23–0.32 A/m^2^). Other factors for safety include stimulation duration, polarity, and electrode shape.

In our current study, the non-significant results suggest no obvious difference from the sham group and reflect that tDCS-iTBS waveform in the range of 2.5 to 4.5 mA/cm^2^ is safe for use in animals. The rationale behind this study was to determine the safety of our newly developed electrical current waveform for brain stimulation. We have reported the same waveform neuroplastic effects in previous research in rats and stroke patients using MEP recording and upper limb physical activity questionnaires, and time to expand tDCS-iTBS waveform outcomes to animal and clinical populations. According to the FDA Medwatch program database, tDCS caused no adverse effects in clinical trials. From this study, we report non-significant results after short- and long-term stimulations in an animal model. These outcomes open new avenues for other researchers to know the safety parameters and translate this stimulation protocol for large-scale animal testing and clinical settings by adjusting dosing parameters without adverse effects.

Based on the results, we conclude that short-term and long-term stimulation did not induce significant adverse effects on brain and skin tissues assessed through biomarkers and histological analysis. The tDCS-iTBS waveform from 2.5 to 4.5 mA/cm^2^ can be used safely for therapeutic purposes in animals to attain stronger neuroplastic effects. In the present study, the current density was lower than the threshold reported in previous studies. It is worth noting that in this study, with 2.5–4.5 mA/cm^2^ intensity, the effect on brain tissue biomarkers and skin/serum markers did not show a significant change from the sham, which proves that these stimulation interventions are safe to use for animal studies without causing brain tissue injury. This stimulation waveform can have applications in clinical use for various neurological diseases to generate neuroplastic effects without brain and scalp tissue lesions.

### Future Research Direction

In the current study, we tried to determine the safety of the tDCS-iTBS waveform in an animal model and employed brain (caspase-3, MBP, and NSE) and scalp tissue injury biomarkers (IL-6) and brain tissue morphology (H&E staining). The genetic biomarkers of localized brain tissue stimulation, joule heating, and polarity of cathodal or anodal stimulation should be researched in future studies. 

## Figures and Tables

**Table 1:** Descriptive statistics of biomarkers during short- and long-term stimulation (*n* = 31).

Subgroups			Sham		Low		High	
Parameters			Mean ± SD	Min–Max	Mean ± SD	Min–Max	Mean ± SD	Min–Max
Groups	Short-term stimulation (*n* = 15)	Caspase-3 (units)	0.39 ± 0.10	0.24–0.50	0.47 ± 0.11	0.34–0.56	0.48 ± 0.08	0.34–0.56
		Cortical MBP (ng/mg)	2.57 ± 0.57	2.10–3.55	2.62 ± 0.44	2.10–3.30	2.71 ± 0.27	2.51–3.18
		Serum MBP (ng/mL)	0.01 ± 0.02	0.00–0.05	0.02 ± 0.02	0.01–0.05	0.01 ± 0.02	0.00–0.05
		Cortical NSE (ng/mg)	5.96 ± 0.69	4.81–6.60	5.86 ± 1.16	3.93–6.80	6.26 ± 0.80	5.48–7.58
		Cortical IL-6 (pg/mg)	1748.81 ± 122.30	1577.53–1902.30	1871.44 ± 332.47	1435.70–2300.69	1985.92 ± 511.68	1275.53–2542.05
		Serum IL-6 (pg/mg)	848.71 ± 368.95	492.57–1436.97	830.49 ± 263.27	535.99–1200.00	888.29 ± 347.84	283.70–1138.80
	Long-term stimulation (*n* = 16)	Caspase-3 (units)	0.47 ± 0.14	0.24–0.72	--	--	0.51 ± 0.21	0.23–0.87
		Cortical MBP (ng/mg)	2.71 ± 0.27	2.34–3.10	--	--	2.87 ± 0.25	2.50–3.20
		Serum MBP (ng/mL)	0.03 ± 0.01	0.01–0.04	--	--	0.02 ± 0.01	0.01–0.04
		Cortical NSE (ng/mg)	6.48 ± 0.72	5.40–7.30	--	--	6.33 ± 1.21	3.90–7.70
		Cortical IL-6 (pg/mg)	2046.38 ± 175.93	1833.00–2432.00	--	--	1930.00 ± 263.71	1576.00–2455.00
		Serum IL-6 (pg/mg)	801.63 ± 213.89	490.00–1200.00	--	--	865.75 ± 502.56	145.00–1600.00

Mean ± SD, average and standard deviation; min, minimum value; max, maximum value; short-term stimulation for 1 h/session (*n* = 15: sham = 5, low = 5, high = 5), long-term stimulation for 3 sessions/week for total 4 weeks (*n* = 16: sham = 8, high = 8), subgroups (low = 2.5 mA/cm^2^, high = 4.5 mA/cm^2^); *n*, number of animals; ng/mg, nanogram per milligram; ng/mL, nanogram per milliliter; pg/mg, picogram per milligram; --, study included three subgroups (sham, low, & high) for short-term stimulation while long-term stimulation has only two subgroups (sham & high).

**Table 2:** Stimulation parameters for short- and long-term stimulations (tDCS-iTBS) (*n* = 31).

Parameters	Short-Term Stimulation (*n* = 15)			Long-Term Stimulation (*n* = 16)		
	Sham	Low	High		Sham	High
Current (mA)	0	2.5	4.5		0	4.5
Current density (mA/cm^2^)	0	2.5	4.5		0	4.5
Charge (C)	0	9	16.2		0	8.1
Charge density (C/m^2^)	0	90,000	162,000		0	81,000
Duration	1 h	1 h	1 h		30 min	30 min
Number of sessions	1 session	1 session	1 session		12 sessions	12 sessions

Short-term (*n* = 15: sham = 5, low = 5, high = 5) and long-term (*n* = 16: sham = 8, high = 8) stimulation consisted of iTBS output from tDCS; iTBS, 1.5 mA; tDCS, 1–3 mA (low =1 mA, high =3 mA); *n*, number of animals; 12 sessions, 3 sessions/week for 4 total weeks.

## Data Availability

Not applicable.
